# Induction of NK Cell Reactivity against B-Cell Acute Lymphoblastic Leukemia by an Fc-Optimized FLT3 Antibody

**DOI:** 10.3390/cancers11121966

**Published:** 2019-12-06

**Authors:** Bastian J. Schmied, Martina S. Lutz, Fabian Riegg, Latifa Zekri, Jonas S. Heitmann, Hans-Jörg Bühring, Gundram Jung, Helmut R. Salih

**Affiliations:** 1 Clinical Collaboration Unit Translational Immunology, German Cancer Consortium (DKTK), Department of Internal Medicine, University Hospital Tübingen, 72076 Tübingen, Germany; Bastian.Schmied@med.uni-tuebingen.de (B.J.S.); Martina.Lutz@med.uni-tuebingen.de (M.S.L.); Fabian.Riegg@med.uni-tuebingen.de (F.R.); l.zekri-metref@dkfz-heidelberg.de (L.Z.); Jonas.Heitmann@med.uni-tuebingen.de (J.S.H.); 2 DFG Cluster of Excellence 2180 ‘Image-guided and Functional Instructed Tumor Therapy’ (iFIT), Eberhard Karls University, 72076 Tübingen, Germany; gundram.jung@uni-tuebingen.de; 3 Department for Immunology, Eberhard Karls University, 72076 Tübingen, Germany; 4 Department of Hematology and Oncology, Eberhard Karls University, 72076 Tübingen, Germany; hans-joerg.buehring@uni-tuebingen.de

**Keywords:** acute lymphoblastic leukemia, B-ALL, immunotherapy, antibody, NK cells, ADCC, FLT3, CD135

## Abstract

Antibody-dependent cellular cytotoxicity (ADCC) is a major mechanism by which antitumor antibodies mediate therapeutic efficacy. At present, we evaluate an Fc-optimized (amino acid substitutions S239D/I332E) FLT3 antibody termed 4G8-SDIEM (FLYSYN) in patients with acute myeloid leukemia (NCT02789254). Here we studied the possibility to induce NK cell ADCC against B-cell acute lymphoblastic leukemia (B-ALL) by Fc-optimized FLT3 antibody treatment. Flow cytometric analysis confirmed that FLT3 is widely expressed on B-ALL cell lines and leukemic cells of B-ALL patients. FLT3 expression did not correlate with that of CD20, which is targeted by Rituximab, a therapeutic monoclonal antibody (mAb) employed in B-ALL treatment regimens. Our FLT3 mAb with enhanced affinity to the Fc receptor CD16a termed 4G8-SDIE potently induced NK cell reactivity against FLT3-transfectants, the B-ALL cell line SEM and primary leukemic cells of adult B-ALL patients in a target-antigen dependent manner as revealed by analyses of NK cell activation and degranulation. This was mirrored by potent 4G8-SDIE mediated NK cell ADCC in experiments with FLT3-transfectants, the cell line SEM and primary cells as target cells. Taken together, the findings presented in this study provide evidence that 4G8-SDIE may be a promising agent for the treatment of B-ALL, particularly in CD20-negative cases.

## 1. Introduction

Cancer immunotherapy with its possibility to elicit a specific antitumor immune reaction has become a mainstay of treatment in many malignancies [1]. In particular, monoclonal antibodies (mAb) are meanwhile well established and have greatly improved the treatment options for patients with malignant diseases. Prominent examples such as Trastuzumab and Rituximab are routinely used for treatment of human epidermal growth factor receptor 2 (HER2)-positive breast cancer and B-cell malignancies, respectively [2,3]. Nevertheless, the therapeutic efficacy of many antitumor mAb still leaves room for improvement. In addition, there are many tumor entities for which so far therapeutic antibodies are not available. A promising strategy to overcome the first problem, that is limited therapeutic efficacy, is to enhance the immunostimulatory potency of a given antibody’s Fc-part [4], particularly by increasing its capacity to induce antibody dependent cellular cytotoxicity (ADCC). The latter represents one of the most important effector mechanisms of such antitumor mAb, at least in blood cancers (e.g., [5]). Enhanced ADCC can be achieved by increasing the affinity of an antibody’s Fc part to the Fcγ receptor IIIa (FcγRIIIa/CD16a) that is expressed by immune cells like natural killer (NK) cells. The latter constitute the most relevant immune cell population that mediates ADCC, at least in humans [6,7]. A frequently pursued approach to increase affinity to CD16a is to optimize the Fc part’s amino acid sequence, e.g., by the amino acid substitutions S239D/I332E (SDIE). This modification increases the Fc-part’s affinity to FcγR in general, but with a more pronounced effect achieved for the activating FcγRIIIa/CD16a compared to the inhibitory FcγRIIb/CD32b [8]. Alternatively, enhanced immunostimulatory efficacy can be achieved by modifications of the Fc-part’s glycosylation pattern. At present, a glyco-engineered CD20 antibody, Obinutuzumab, is approved for treatment of certain B-cell malignancies, and many mAb with amino acid substitutions in their Fc part are being tested in clinical trials [4].

Besides ADCC-inducing antitumor mAb, other antibody-based approaches are meanwhile approved to treat B-cell acute lymphoblastic leukemia (B-ALL). Immunotherapeutic strategies like the CD19xCD3 bispecific T cell engager (BiTE) Blinatumomab or the anti-CD19 CAR-T cell product Tisagenlecleucel mediate impressive effects upon treatment of relapsed/refractory (r/r) B-ALL [9,10]. Furthermore, an antibody drug conjugate (ADC) termed Inotuzumab ozogamicin aiming for targeted cytotoxic drug delivery to CD22 expressing B-ALL cells has been proven to be superior to standard therapy in r/r B-ALL [11]. Presently ongoing clinical studies such as NCT03628053 hopefully will unravel the benefits and risks of CAR-T cell therapy compared to Blinatumomab and Inotuzumab ozogamicin.

We have recently introduced mAb and antibody-related constructs carrying the SDIE modification for immunotherapy of different leukemic and solid tumor entities [12,13,14,15,16,17,18]. The most advanced of our compounds is an Fc-optimized mAb, which targets FMS-like tyrosine kinase 3 (FLT3) expressed on the cell surface of leukemic cells in the vast majority of patients with acute myeloid leukemia (AML). At present, this construct termed 4G8-SDIEM (FLYSYN) is undergoing clinical evaluation in a phase I study enrolling AML patients with the aim to eliminate minimal residual disease (NCT02789254). Notably, beyond AML, FLT3 has also been reported to be expressed in B-ALL [19]. Accordingly, we here set out to characterize the suitability of targeting FLT3 with an Fc-optimized mAb for treatment of B-ALL with the aim to expand the usage and benefits of mAb treatment also to the ~70% of B-ALL patients that do not display CD20 expression on leukemic cells [20].

## 2. Results

### 2.1. FLT3 Surface Expression on B-ALL Cell Lines and Primary Cells as Recognized by the FLT3 Binder 4G8

**Figure 1:**
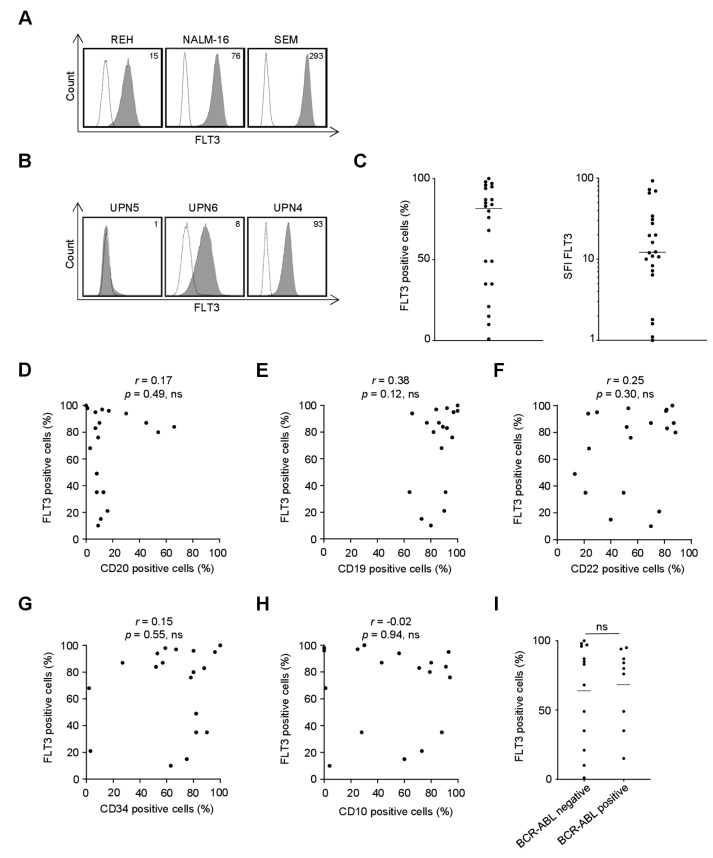
Recognition of FLT3 expressed on the surface of B-cell acute lymphoblastic leukemia (B-ALL) cell lines and primary cells by mAb 4G8. B-ALL cell lines and primary leukemic cells of B-ALL patients were incubated with mouse anti-human FLT3 mAb clone 4G8 or murine IgG1 as isotype control (both 10 µg/mL) followed by a goat anti-mouse PE conjugate and subsequently analyzed by flow cytometry. (**A**) Exemplary data for FLT3 expression on REH, NALM-16 and SEM cells is shown (shaded peaks, anti-FLT3; open peaks, control). Numbers in the upper right corner depict specific fluorescence intensity (SFI) levels calculated as described in the method section. (**B**,**C**) Malignant cells within peripheral blood mononuclear cells of B-ALL patients (*n* = 22) were identified by counterstaining for CD34, CD10, CD19 or CD20 according to their pre-specified immunophenotype. (**B**) Exemplary data for patient cells with no (left), intermediate (middle) and high (right) surface expression of FLT3. (**C**) Combined analysis with FLT3 surface expression depicted as % FLT3^+^ B-ALL blasts (left) and SFI levels (right). (**D**–**I**) Association of FLT3 surface expression (depicted as % positive cells) on primary B-ALL samples with expression of CD20 (**D**), CD19 (**E**), CD22 (**F**), CD34 (**G**), CD10 (**H**) and BCR-ABL (**I**). *p*: *p*-value; *r*: Pearson correlation coefficient; UPN: uniform patient number.

As a first step, we characterized whether and to what extent the specific FLT3 antibody clone used for generation of our Fc-optimized mAb recognizes FLT3 on the surface of B-ALL cells. To this end, three commonly used B-ALL cell lines as well as primary leukemic cells from a cohort of 22 adult B-ALL patients were analyzed by flow cytometry. The clinical characteristics of the patients are depicted in Table S1. As shown in Figure 1A–C, FLT3 is widely expressed in B-ALL, however to a highly variable extent. Substantial expression (≥20% surface expression) as detected by the FLT3 mAb 4G8, the specific antibody clone used for generation of 4G8-SDIE undergoing evaluation for B-ALL treatment in this study, was observed in all three cell lines and in 86% (19 of 22) of the investigated patient samples. We then determined whether FLT3 expression on primary leukemic cells correlated with other molecules reportedly involved in B-ALL disease pathophysiology or serving as targets for therapeutic approaches (Figure 1D–I; Table S1). We observed a trend for a correlation between FLT3 and CD19 expression that failed to reach statistical significance, while no obvious correlation was observed for FLT3 and expression of CD20, CD22, CD34 and CD10 as well as positivity for BCR-ABL. While studies in larger cohorts are required to comprehensively study potential correlations, the lack of a clear association of FLT3 expression with the prevalence of other therapeutic targets like CD19, CD20, CD22, BCR-ABL and the high binding capacity of antibody clone 4G8 in our view substantiate the value of FLT3 targeting by 4G8-SDIE for treatment of B-ALL.

### 2.2. Production and Characterization of 4G8-SDIE

**Figure 2:**
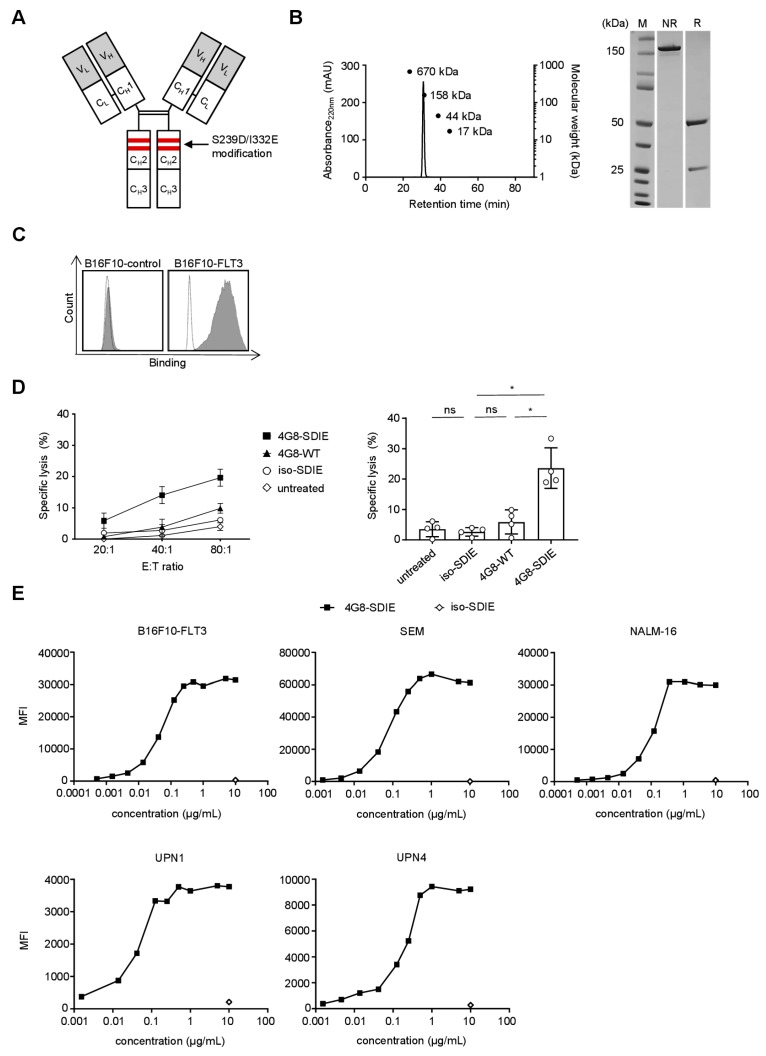
Production and characterization of 4G8-SDIE. (**A**) Schematic illustration of the structure of 4G8-SDIE. (**B**) Purified 4G8-SDIE was analyzed by size exclusion chromatography (left) and SDS-PAGE (right). Expected molecular weights, based on the amino acid sequence, were ~23, ~49 and ~145 kDa for light chain, heavy chain and full antibody, respectively. Dots represent the respective standards. M: marker; mAU: milli absorption unit; NR: non-reduced; R: reduced. (**C**) B16F10-FLT3 or control transfectants were incubated with 5 µg/mL 4G8-SDIE or iso-SDIE (isotype control antibody with similar characteristics, but irrelevant target specificity) followed by an anti-human phycoerythrin (PE) conjugate and analyzed by flow cytometry. Shaded peaks: 4G8-SDIE; open peaks: iso-SDIE. (**D**) Peripheral blood mononuclear cells (PBMC) of healthy donors were cultured with primary B-ALL cells in the presence or absence of iso-SDIE, chimeric 4G8 with wildtype Fc-part (4G8-WT) or 4G8-SDIE (all 10 µg/mL). B-ALL cell lysis was analyzed by 2 h Europium cytotoxicity assays. On the left, exemplary results obtained with cells from one healthy PBMC donor and one B-ALL patient are shown, on the right pooled data obtained with cells from two PBMC donors and B-ALL patients UPN4/6 at an E:T ratio of 80:1 are depicted. Bars and error bars represent means of results and standard deviations, respectively. (**E**) B16F10-FLT3 transfectants, the B-ALL cell lines SEM and NALM-16, and primary cells of two B-ALL patients (UPN 1 and 4) were incubated with increasing concentrations of 4G8-SDIE or iso-SDIE (10 µg/mL) followed by an anti-human PE conjugate and analyzed by flow cytometry. Malignant cells within PBMC of B-ALL patients were identified according to their pre-specified immunophenotype. Mean fluorescence intensities (MFI) are depicted. *: significant (*p*-value < 0.05); ns: not significant; UPN: uniform patient number.

The murine anti-FLT3 clone 4G8 was chimerized (human immunoglobulin G1/Κ constant region) and Fc-optimized (S239D/I332E modification) as described in the methods section (Figure 2A). Note that the resulting construct termed 4G8-SDIE slightly differs from 4G8-SDIEM (FLYSYN) that is presently undergoing clinical evaluation in AML as it does not contain an M-tag. The latter was originally introduced for better detection in human sera [12], which meanwhile can be achieved by other technical means. Notably, presence or absence of the M-tag does not affect target antigen binding or NK cell stimulatory properties (Figure S1).

Our construct 4G8-SDIE is produced with good yield, and analysis by size exclusion chromatography and SDS-PAGE confirmed the expected molecular weights as well as the protein’s purity and lack of aggregates (Figure 2B). Flow cytometric analyses using B16F10-FLT3 and control transfectants confirmed that 4G8-SDIE specifically bound to its target antigen FLT3 (Figure 2C). Next we conducted Europium cytotoxicity assays with healthy peripheral blood mononuclear cells (PBMC) as effector cells and primary B-ALL cells as targets. In line with our previously published findings regarding the efficacy of 4G8-SDIEM with the B-ALL cell line NALM-16 [12], 4G8-SDIE induced superior target cell lysis when compared to its chimeric counterpart containing a wildtype Fc-part (Figure 2D and Figure S2). Binding titration experiments using B16F10-FLT3 transfectants as well as the B-ALL cell lines NALM-16 and SEM—the latter displaying the highest FLT3 expression in our study—revealed that 1 µg/mL 4G8-SDIE was sufficient for saturating target antigen binding. This was confirmed with primary leukemic cells of B-ALL patients UPN1 and UPN4 (Figure 2E).

### 2.3. Induction of NK Cell Reactivity against FLT3^+^ Target Cells

**Figure 3:**
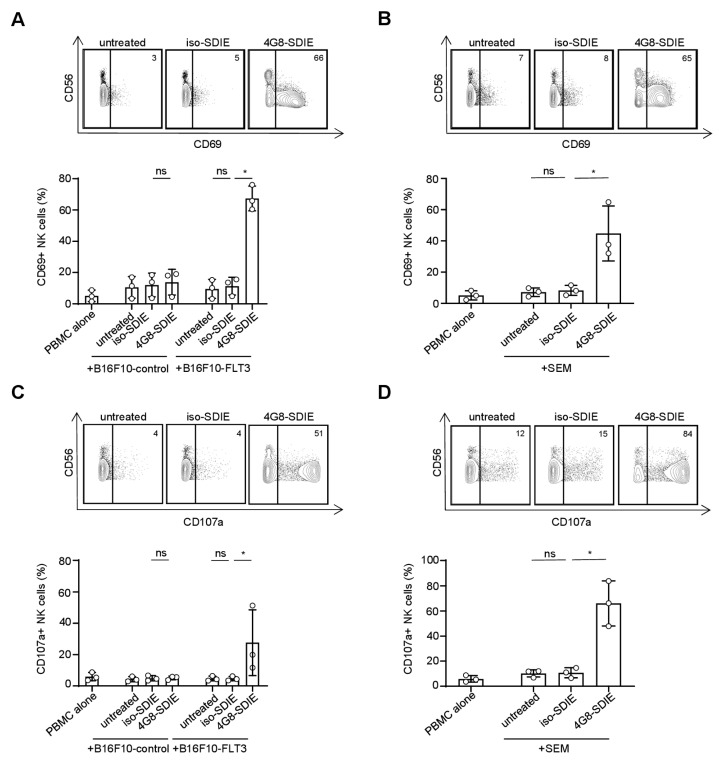

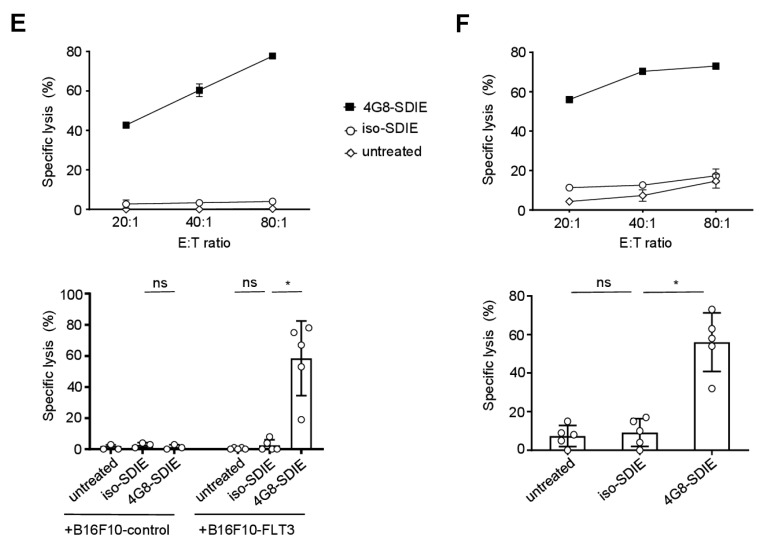
Induction of natural killer (NK) cell reactivity against FLT3^+^ target cells. Peripheral blood mononuclear cells (PBMC) of healthy donors were cultured with or without B16F10-FLT3 or control transfectants (**A**,**C**,**E**) or the FLT3^+^ B-ALL cell line SEM (**B**,**D**,**F**) in the presence or absence of 4G8-SDIE/iso-SDIE (1 µg/mL). Top panels of each subfigure display exemplary data obtained with PBMC from one donor and B16F10-FLT3 or SEM cells. Bottom panels depict combined results from analyses with three to five independent PBMC donors. Bars and error bars represent means of results and standard deviations, respectively. (**A**,**B**) Cells were cultured at an effector to target (E:T) ratio of 2.5:1 for 24 h. Subsequently, activation of NK cells identified as CD19^−^CD56^+^CD3^−^ lymphocytes was determined by flow cytometric analysis of CD69. (**C**,**D**) Cells were cultured at an E:T ratio of 2.5:1 for 4 h in the presence of GolgiStop, GolgiPlug and an anti-human CD107a phycoerythrin (PE) conjugate. Subsequently, degranulation of NK cells (CD19^−^CD56^+^CD3^−^ lymphocytes) was determined by flow cytometric analysis of CD107a. (**E**,**F**) Target cell lysis was analyzed by 2 h Europium cytotoxicity assays. Combined analyses show data obtained at an E:T ratio of 80:1. ns: not significant; *: significant (*p*-value < 0.05).

Next we analyzed whether 4G8-SDIE induced an immune response specifically against FLT3^+^ tumor cells. To this end, PBMC of healthy donors were cultured with B16F10-FLT3 or control transfectants in the presence or absence of 4G8-SDIE. We found that 4G8-SDIE potently induced NK cell reactivity as revealed by upregulation of the NK cell activation and degranulation markers CD69 and CD107a, respectively, as well as target cell lysis in the presence of B16F10-FLT3 transfectants. No effects were observed with an isotype control mAb termed iso-SDIE or when the FLT3^−^ control transfectants were used as targets, which confirmed the target-antigen restricted efficacy of 4G8-SDIE (Figure 3A,C,E and Figure S3A). The same experimental systems were then utilized employing the FLT3^+^ B-ALL cell line SEM as target cells. In line with the previous findings, 4G8-SDIE was found to specifically and potently induce NK cell activation, degranulation and lysis of the B-ALL cell line SEM (Figure 3B,D,F and Figure S3B).

### 2.4. Induction of NK Cell Immunity Against Primary B-ALL Cells

**Figure 4:**
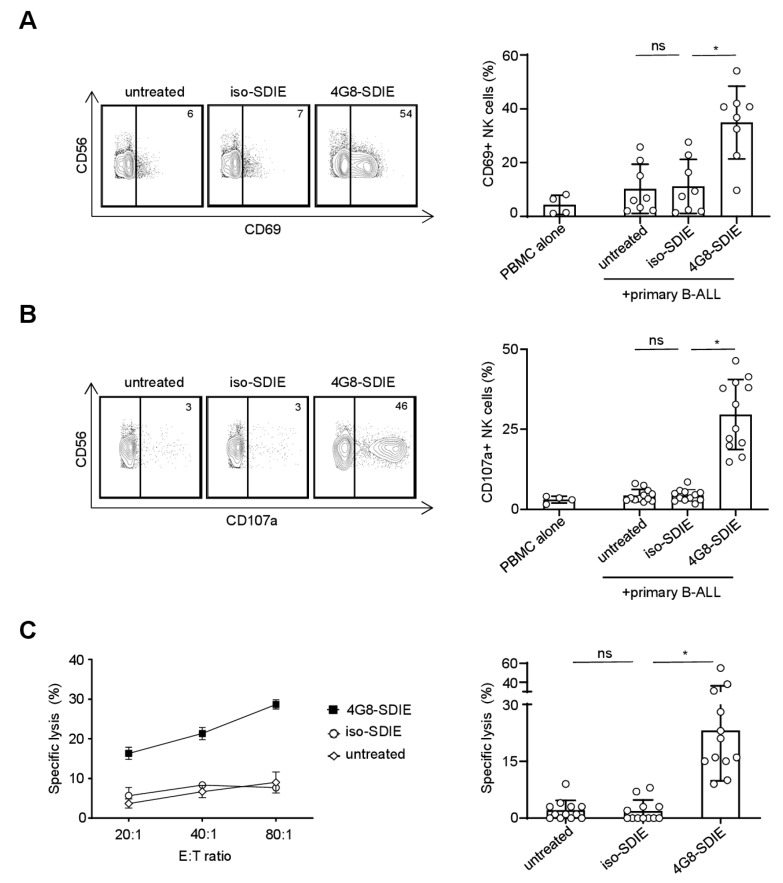
Induction of NK cell reactivity against primary B-ALL cells. Peripheral blood mononuclear cells (PBMC) of healthy donors were cultured with or without FLT3^+^ B-ALL patient cells (UPN1/4/6/12/17/22, all blast count ≥86%) in the presence or absence of 4G8-SDIE/iso-SDIE (1 µg/mL). Left panels depict exemplary results obtained with cells from one healthy PBMC donor and one B-ALL patient; right panels show combined results obtained in multiple analyses with cells from different PBMC donors and B-ALL patients. Bars and error bars represent means of results and standard deviations, respectively. (**A**) Cells were cultured at an effector to target (E:T) ratio of 2.5:1 for 24 h. Subsequently, activation of NK cells identified as CD19^−^CD56^+^CD3^−^ lymphocytes was determined by flow cytometric analysis of CD69. Combined analyses show data obtained with cells from two healthy PBMC donors and four B-ALL patients. (**B**) Cells were cultured at an E:T ratio of 2.5:1 for 4 h in the presence of GolgiStop, GolgiPlug and an anti-human CD107a phycoerythrin (PE) conjugate. Subsequently, degranulation of NK cells (CD19^−^CD56^+^CD3^−^ lymphocytes) was determined by flow cytometric analysis of CD107a. Combined analyses show data obtained with cells from two healthy PBMC donors and six B-ALL patients. (**C**) B-ALL cell lysis was analyzed by 2 h Europium cytotoxicity assays. On the left exemplary data obtained with cells from one healthy PBMC donor and one B-ALL patient at different E:T ratios, on the right pooled data obtained with cells from three healthy PBMC donors and five B-ALL patients at an E:T ratio of 80:1 are shown. ns: not significant; *: significant (*p*-value < 0.05).

We next determined the capacity of 4G8-SDIE to induce NK cell reactivity against primary leukemic cells of B-ALL patients. To this end, PBMC of healthy donors were cultured with FLT3^+^ leukemic cells from adult B-ALL patients in the presence or absence of 4G8-SDIE or iso-SDIE. Flow cytometric analysis of CD69 expression confirmed that 4G8-SDIE potently induced NK cell activation, whereas the control mAb had no effect (Figure 4A). Likewise, determination of CD107a upregulation revealed that 4G8-SDIE potently induced NK cell degranulation (Figure 4B). Finally, the efficacy of 4G8-SDIE to mediate lysis of primary B-ALL cells was comprehensively studied by Europium cytotoxicity assays, which confirmed that 4G8-SDIE indeed was capable to induce NK cell ADCC against primary leukemic cells of B-ALL patients (Figure 4C and Figure S4).

## 3. Discussion

We here reported on the characterization of an Fc-optimized mAb targeting the receptor tyrosine kinase FLT3 for treatment of B-ALL. FLT3 is widely expressed on leukemic cells in AML, and at present a clinical study is conducted in which we evaluate the Fc-optimized FLT3 mAb 4G8-SDIEM (FLYSYN) to induce NK cell reactivity in AML patients with minimal residual disease (NCT02789254). Beyond AML, FLT3 reportedly is also expressed in B-ALL (e.g., [19]), which led us to reason that targeting FLT3 might constitute a promising immunotherapeutic strategy also for this disease. The Fc-optimized FLT3 mAb 4G8-SDIE that we evaluated in this study differs from 4G8-SDIEM by the fact that it lacks an M-tag. The M-tag was omitted in the present construct as meanwhile other methods are available for detection of 4G8-SDIE in human serum. Notably, the lack of the M-tag does neither affect target antigen binding nor NK cell stimulatory properties. After demonstrating that 4G8-SDIE can bind primary B-ALL cells in 86% of cases at clinically achievable concentrations, we used various experimental systems to demonstrate that 4G8-SDIE indeed potently induces NK cell reactivity against B-ALL cells in a target-antigen dependent manner.

While optimized multi-agent chemotherapy regimens resulted in high cure rates of ~90% in pediatric ALL, cure rates so far remain as low as ~40% in adult ALL [21]. This underlines the pressing need for novel therapies and holds particularly true for adult B-ALL, which was at the focus of the present study. Targeting of BCR-ABL, which is present in 15–25% of adult B-ALL patients, by tyrosine inhibitors such as Imatinib, Dasatinib and Ponatinib clearly improved the outcome for the respective patients [22]. In addition, the introduction of antibody-based approaches has improved therapy options in B-ALL. Presently approved antibody-based strategies comprise the CD19xCD3 bispecific mAb Blinatumomab, the CD22-targeting ADC Inotuzumab ozogamicin and the anti-CD19 CAR T-cell product Tisagenlecleucel. However, while these drugs exert significant therapeutic effects, their activity is still limited, among others due to resistance mechanisms, which are for example associated with the modulation of their target antigens [23]. Downmodulation of target antigens associated with failure of therapy has been reported for approaches comprising binders of CD19 like the BiTE Blinatumomab and also anti-CD19 CAR-T cells [24,25] as well as the CD22-targeting ADC Inotuzumab ozogamicin [26,27,28]. With our FLT3 binder 4G8, we also observed downmodulation of FLT3 expression on AML and B-ALL cells [12], but even high mAb concentrations did not reduce antigen expression by more than 30%–40%. Due to their generally lower toxicity in comparison to T-cell immunotherapeutics or ADC, in particular ADCC-inducing mAb could allow for combination treatment in order to target multiple antigens and reduce the risk of therapy failure due to antigen escape of a single antigen. Widely used “off-label” in therapeutic regimes for CD20 positive B-ALL is the anti-CD20 mAb Rituximab, which enabled significant improvements of outcome when added to chemotherapy [29]. Notably, Rituximab was shown to elicit its beneficial effects in great part through the induction of NK cell ADCC [5]. However, only ~30% of B-ALL patients express CD20 (≥20% surface expression; [20]), which certainly constitutes a drawback for the use of Rituximab and lends support to our reasoning that 4G8-SDIE might prove useful in this disease.

Given the importance of ADCC induction for the efficacy of antitumor mAb, particularly in hematological malignancies [6,7], many efforts presently aim to increase the efficacy of antitumor mAb by enhancing the affinity of the Fc part to CD16a expressed, e.g., on NK cells and thus improve ADCC. This can be achieved by optimizing the Fc-part’s glycosylation pattern as exemplified by the CD20 mAb Obinutuzumab, which is approved for treatment of chronic lymphocytic leukemia [30] and also showed superior in vitro results with B-ALL cells when compared to Rituximab [31]. Improved ADCC induction can furthermore be accomplished by changes in the Fc-part’s amino acid sequence such as the substitutions S239D/I332E (SDIE modification) that are contained in 4G8-SDIE. In line, superior NK cell ADCC against primary B-ALL cells compared to its counterpart with wildtype Fc-part was observed with 4G8-SDIE, which is also in agreement with results described in our report on 4G8-SDIEM in AML [12] and findings of other investigators [8]. Besides constructs targeting FLT3, many other Fc-engineered mAb directed to other antigens that carry the SDIE modification are presently evaluated in clinical trials. This includes, e.g., the anti-HER2 mAb Margetuximab (NCT01828021), the anti-CD19 mAb Tafasitamab (NCT01685021), the anti-CD157 mAb MEN1112 (NCT02353143) and the anti-CD33 mAb BI 836858 (NCT02240706, NCT03013998).

The fact that FLT3 is more frequently expressed in B-ALL than CD20 underlines the potential of this target antigen in this disease. Notably, the analyses with primary B-ALL samples in our study, which revealed a potent induction of NK cell ADCC by 4G8-SDIE, included several CD20 negative cases. This provides further evidence for the value of FLT3 targeting in B-ALL, in particular since other investigators implicated FLT3 to be expressed on leukemic stem cells in ALL [32] and that FLT3 may associate with B-ALL cell resistance to conventional therapy [33]. Heterogeneous expression of FLT3 on the mRNA level and on the cell surface in B-ALL patients has been reported by other investigators [34,35,36], and a trend for a correlation of low FLT3 expression and relapse was observed in an Indian cohort of ALL patients, without reaching statistical significance [34]. In contrast, in patients with mixed-lineage leukemia (MLL)-rearranged infant ALL, which per se has a poor prognosis, high FLT3 expression was associated with a significantly shortened event-free survival [35]. Also another group observed high FLT3 expression especially in MLL-rearranged ALL, and high FLT3 expression was identified as independent prognostic marker for shorter overall survival and poorer treatment outcome in this particular subgroup of B-ALL patients [36]. In future studies, we plan to investigate the reactivity of 4G8-SDIE specifically in subfractions of B-ALL cells and in patient groups with dismal prognosis.

Notably, beyond 4G8-SDIE, also other therapeutic approaches aim at targeting surface expressed FLT3 to induce antitumor immunity. These comprise CAR NK and T cells [37,38], bispecific FLT3xCD3 antibodies [39], the complement-dependent cytotoxicity inducing FLT3 mAb A2 [40] and the FLT3 mAb IMC-EB10 that was intended to induce NK cell ADCC [41]. All of these targeting strategies vary widely in numerous aspects, e.g., therapeutic efficacy, toxicities and mechanisms of resistance, which will have to be addressed in further studies to define the best FLT3-targeting approach. Notably, this will also require clinical evaluation, as highlighted by the results of the clinical phase I study (NCT00887926) evaluating the FLT3 mAb IMC-EB10 in AML [41]. The study was not successful, possibly due to the fact that IMC-EB10 was not optimized for ADCC induction, which is supported by our comparative analyses of NK cell ADCC using 4G8-SDIE and its chimeric counterpart with wildtype Fc-part. When considering adoptively transferred CAR T or NK cells, it should be considered that products like Tisagenlecleucel and Axicabtagene Ciloleucel require a personalized approach resulting in manufacturing times of 3–4 weeks. This in turn leads to treatment delay and high costs. Antibodies, in contrast, are readily available drugs and thus 4G8-SDIE would represent a universal, “off-the shelf” product.

Besides efficacy, toxicity is an important aspect in the development of therapeutic mAb. Exploring potential “on-target off-tumor” toxicity, we did not detect binding of our FLT3 binder 4G8 to a wide array of normal cryopreserved tissues as well as to thrombocytes, erythrocytes and granulocytes in immunohistological and flow cytometric analyses, respectively [12]. However, 4G8 weakly binds to healthy hematopoietic progenitor cells as well as dendritic cells as observed by flow cytometry. In comparison with leukemic cells, however, FLT3 is expressed at very low level on these healthy cells (about 500–600 and <300 molecules/cell, respectively). This low antigen density did not result in unwanted effects of 4G8-SDIEM against these populations in different experimental systems in our antecedent study. Of note, “on-target off-tumor” toxicity against healthy FLT3^+^ cells were indeed observed with FLT3xCD3 bispecific antibodies that stimulate T cells, as well as with CAR-T cells; for both, such low target antigen levels are sufficient to induce activation [12,38,39]. 4G8-SDIE yielded saturated FLT3 binding and showed potent efficacy against target cells including primary B-ALL cells at 1 µg/mL. We assume that such drug levels are easily achieved in B-ALL, as even doses of 45 mg/m^2^ 4G8-SDIEM did not cause overt and dose-limiting side effects in our clinical trial in AML patients ([42], study results to be presented at the ASH meeting 2019). Of note, this dosing is more than 2–3 orders of magnitude higher than routine dosing, e.g., of the CD19xCD3 BiTE Blinatumomab. Besides the higher effector potential of T cells compared to NK cells that contributes to this discrepancy, BiTE antibodies like Blinatumomab show a tendency to aggregation that may result in unspecific “off target off tumor” immune activation, which in turn limits applicable doses [43,44,45]. The favorable safety profile of our Fc-optimized FLT3 mAb could thus be particularly important in elderly and frail patients, which are not eligible for chemotherapy or treatment with Blinatumomab due to the potential serious toxicities [46].

In conclusion, the analyses reported in this study regarding the efficacy of 4G8-SDIE in B-ALL, together with the available data on the favorable safety profile upon application of Fc-optimized FLT3 mAb to AML patients, in our view clearly indicate that 4G8-SDIE constitutes a promising immunotherapeutic compound for treatment of B-ALL.

## 4. Materials and Methods

### 4.1. Production, Purification and Structural Analysis of Fc-Optimized Antibodies

4G8-SDIE and iso-SDIE were generated by chimerization (human immunoglobulin G1/Κ constant region) and Fc-optimization (S239D/I332E modification) of the anti-FLT3 mAb 4G8 and control mAb MOPC21, respectively [12,18]. 4G8-SDIE slightly differs from 4G8-SDIEM (FLYSYN) by the fact that it does not contain an M-tag. In brief, plasmids for the respective heavy and light chains were obtained using the EndoFree Plasmid Maxi kit from Qiagen (Hilden, Germany) according to the manufacturer’s recommendations. Antibodies were produced in ExpiCHO cells (Gibco, Carlsbad, CA, USA) according to the manufacturer’s recommendations and purified by affinity (Mabselect; GE Healthcare, Chicago, IL, USA) as well as subsequent preparative size exclusion chromatography (HiLoad 16/60 Superdex 200; GE Healthcare). Before use in functional experiments, mAb were cleared of endotoxins with the Endotrap HD Kit from Hyglos (Bernried, Germany). For structural analyses, mAb were investigated by analytical size exclusion chromatography (Superdex 200 Increase 10/300 GL; GE Healthcare) and SDS-PAGE (4%–12% gradient gels; Invitrogen, Carlsbad, CA, USA) using the gel filtration and Precision Plus standards from Bio-Rad (Hercules, CA, USA), respectively. The chimeric version of 4G8 with wildtype Fc part (4G8-WT) was described in [12].

### 4.2. Cells

B16F10-FLT3 and B16F10-control cells were generated by transfecting B16F10 cells (American Type Culture Collection, Manassas, VA, USA) with pcDNA™3.1 based vectors coding for human FLT3 (accession no. NM_004119.2) or CD133 (accession no. BC012089.1), respectively. Transfected cell lines were maintained in DMEM selection medium containing 1 mg/mL G418 (Biochrom, Berlin, Germany).

The B-ALL cell lines NALM-16, REH as well as SEM were obtained from the German Collection of Microorganisms and Cell Cultures (Braunschweig, Germany) and maintained in RPMI1640 as well as IMDM (Gibco) media, respectively. Cell line authenticity was routinely determined by validating the respective immunophenotype described by the provider using flow cytometry, and cells were cultured for a maximum of two months prior to use in experiments. Contamination with mycoplasma was excluded by routine testing of all cultures every three months.

Peripheral blood samples of adult B-ALL patients were obtained at the time of diagnosis. The study was approved by the ethics committee at the Medical Faculty of the Eberhard Karls University and the University Hospital Tübingen (reference number 13/2007V). Human material was collected after obtaining informed consent in accordance with the Helsinki protocol. PBMC from patient samples as well as thrombophoresis products of healthy volunteers were isolated by density gradient centrifugation (Biocoll; Biochrom) and stored in liquid nitrogen. After thawing, PBMC of healthy donors were cultivated in RPMI1640 for 18–24 h prior to use in functional experiments.

All above-mentioned media contained L-Glutamine and were supplemented with 10% heat-inactivated fetal calf serum (Biochrom) and 1% penicillin/streptomycin, DMEM additionally with 1% sodium pyruvate (Lonza, Verviers, Belgium). All cells were maintained at 37 °C and 5% CO_2_ in a humidified atmosphere.

### 4.3. Flow Cytometry

For studies on FLT3 surface expression and 4G8-SDIE binding, cells were stained with the respective unconjugated antibodies or isotype controls followed by species-specific PE conjugates. The murine anti-human FLT3 clone 4G8 was produced as described previously [12]. The goat anti-mouse and donkey anti-human PE conjugates were from Dako (Glostrup, Denmark) and Jackson ImmunoResearch (West Grove, PA, USA), respectively. Leukemic cells within PBMC of B-ALL patients were identified by counterstaining with fluorescently labeled mAb against CD10, CD34, CD19 or CD20 from BD Pharmingen (San Diego, CA, USA) and Biolegend (San Diego, CA, USA) according to their prespecified immunophenotype. The anti-CD22 FITC conjugate was obtained from BD Pharmingen.

For studies on NK cell activation and degranulation, the fluorescently labeled mAb CD69-PE, CD107a-PE from BD Pharmingen as well as CD56-APC, CD3-APC/Fire750 and CD19-BV421 from Biolegend were used.

Corresponding isotype controls were obtained from BD Pharmingen or Biolegend. Dead cells were excluded from analysis by staining with 7-AAD (Biolegend). Measurements were performed using a FACS Canto II or FACS Fortessa (BD Biosciences, San Diego, CA, USA) and data analyzed using the software FlowJo (FlowJo LCC, Ashland, OR, USA). Where stated, specific fluorescence intensity (SFI) levels were calculated by dividing the mean fluorescence intensity (MFI) obtained with specific mAb by the MFI obtained with the respective isotype controls.

Results on expression of CD20, CD19, CD34, CD10 in B-ALL patients were obtained by the flow cytometry diagnostic laboratory of the University Hospital Tübingen according to standard procedures at diagnosis.

### 4.4. Analysis of NK Cell Activation and Degranulation

PBMC of healthy donors were cultured with or without the indicated target cells at an effector to target ratio of 2.5:1 in the presence or absence of 4G8-SDIE/iso-SDIE (1 µg/mL). CD69 expression on NK cells identified as CD19^−^CD56^+^CD3^−^ lymphocytes after 24 h was analyzed by flow cytometry. For studies on NK cell degranulation, cells were cultured for 4 h in the presence of anti-CD107a-PE, BD GolgiStop and BD GolgiPlug (BD Biosciences). Subsequently, NK cells identified as mentioned above were analyzed by flow cytometric determination of CD107a.

### 4.5. Analysis of NK Cell Cytotoxicity 

Target cell lysis by PBMC of healthy donors in the presence or absence of 4G8-SDIE/iso-SDIE (1 µg/mL) was determined by 2 h Europium cytotoxicity assays as previously described [15]. Specific lysis rates were calculated as follows:

100 × (experimental release − spontaneous release)/(maximum release − spontaneous release).

If not indicated otherwise, lysis rates are depicted as means of technical triplicates with standard deviation.

### 4.6. Statistics

The software GraphPad Prism 8 (GraphPad Software, San Diego, CA, USA) was used for statistical analyses. The 95% confidence level was applied. For correlation analyses of surface markers a Pearson correlation, in the case of BCR-ABL an unpaired *t*-test was performed. With regard to functional data, *p*-values were calculated by one-way ANOVA and subsequent Tukey’s multiple comparison tests for normally distributed data. In case of non-normal distribution, *p*-values were calculated by Friedman and subsequent Dunn’s multiple comparisons tests. Where indicated, significantly (*p* < 0.05) and not significantly results between two groups are marked by “*” and “ns”, respectively.

## 5. Conclusions

This study described the characterization of 4G8-SDIE, an Fc-optimized FLT3 antibody, for induction of NK cell reactivity against B-ALL. Based on the observed efficacy and the assumed favorably safety profile, we conclude that 4G8-SDIE constitutes a promising immunotherapeutic compound for treatment of B-ALL that warrants further development.

## Supplementary Materials

The following supplementary materials are available online at https://www.mdpi.com/2072-6694/11/12/1966/s1. Figure S1: Comparison of 4G8-SDIE and 4G8-SDIEM. Figure S2: Enhanced NK cell ADCC against primary B-ALL cells by the Fc-optimized antibody 4G8-SDIE. Figure S3: Induction of NK cell reactivity against FLT3^+^ target cells. Figure S4: Induction of NK cell reactivity against primary B-ALL cells. Table S1: Clinical characteristics of B-ALL patients and FLT3 surface expression levels.
