# Supplementary material for: Induction of NK Cell Reactivity against B-Cell Acute Lymphoblastic Leukemia by an Fc-Optimized FLT3 Antibody
Source: Cancers (Basel). 2019 Dec 6;11(12):1966. doi: 10.3390/cancers11121966 (PMC6966676; doi:10.3390/cancers11121966)
Supplement: Supplementary file 1 [file cancers-11-01966-s001.pdf]

Supplementary Materials

Induction of NK Cell Reactivity against B-Cell Acute Lymphoblastic Leukemia by an Fc-Optimized FLT3 Antibody

Bastian J. Schmied [1,2,†], Martina S. Lutz [1,2,†], Fabian Riegg [1,2], Latifa Zekri [1,2,3], Jonas S. Heitmann [1,2], Hans-Jörg Bühring [4], Gundram Jung [2,3,‡] and Helmut R. Salih [1,2,*,‡]

Supplementary Figures

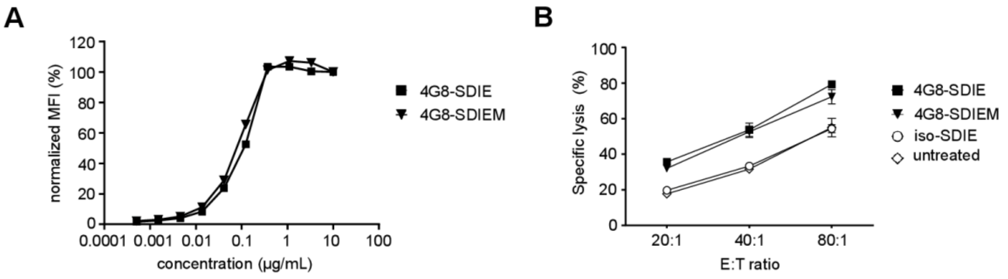

Figure S1. Comparison of 4G8-SDIE and 4G8-SDIEM. (**A**) Cells of the B-ALL cell line NALM-16 were incubated with increasing concentrations of 4G8-SDIE and 4G8-SDIEM followed by a donkey anti-human phycoerythrin (PE) conjugate. Subsequently, binding was analyzed by flow cytometry. Mean fluorescence intensities (MFI) normalized to the MFI of the respective antibody's highest concentration are depicted. (**B**) Lysis of NALM-16 cells by peripheral blood mononuclear cells of a healthy donor in the presence or absence of the indicated antibodies at the indicated effector to target (E:T) ratios was analyzed by a 2 h Europium cytotoxicity assay.

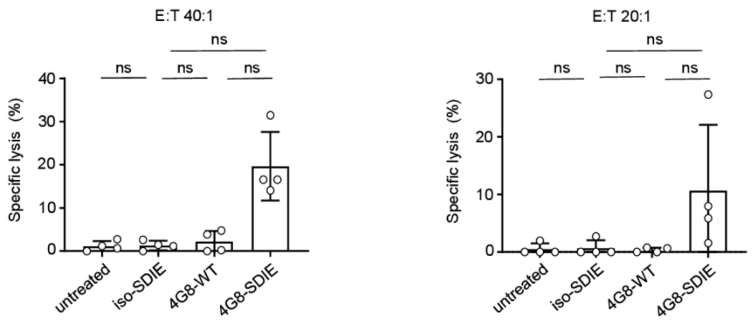

Figure S2. Enhanced NK cell ADCC against primary B-ALL cells by the Fc-optimized antibody 4G8-SDIE. Peripheral blood mononuclear cells (PBMC) of healthy donors were cultured with primary B-ALL cells in the presence or absence of iso-SDIE as control, chimeric 4G8 with wildtype Fc-part (4G8-WT) or 4G8-SDIE (all 10 µg/mL). B-ALL cell lysis was analyzed by 2 h Europium cytotoxicity assays. Pooled data obtained with cells from two healthy PBMC donors and B-ALL patients UPN4/6 at the effector to target (E:T) ratios of 40:1 (left) and 20:1 (right) are depicted. Bars and error bars represent means of results and standard deviations, respectively. ns: not significant (p-value > 0.05).

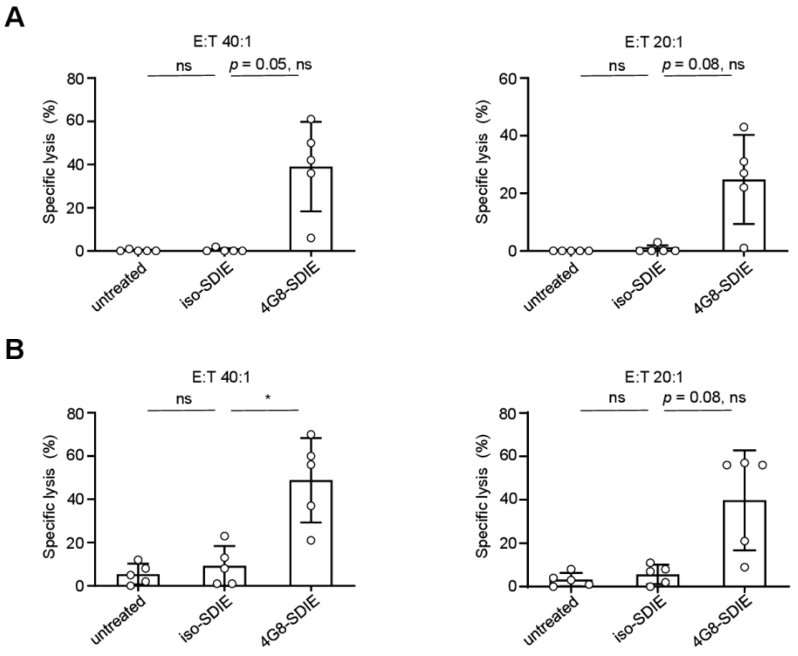

Figure S3. Induction of NK cell reactivity against FLT3+ target cells. Peripheral blood mononuclear cells (PBMC) of five healthy donors were cultured with B16F10-FLT3 transfectants (**A**) or the FLT3+ B-ALL cell line SEM (**B**) at the indicated effector to target (E:T) ratios in the presence or absence of 4G8-SDIE/iso-SDIE (1 μg/mL). Bars and error bars represent means of results and standard deviations, respectively. *p*: *p*-value; ns: not significant; *: significant (*p*-value < 0.05).

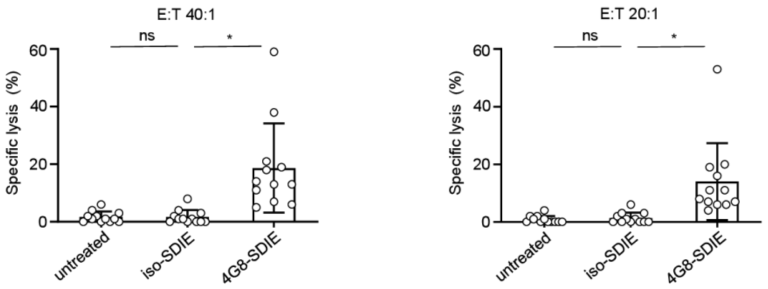

Figure S4. Induction of NK cell reactivity against primary B-ALL cells. Peripheral blood mononuclear cells (PBMC) of healthy donors were cultured with or without $FLT3^+$ B-ALL patient cells in the presence or absence of 4G8-SDIE/iso-SDIE (1 µg/mL). B-ALL cell lysis was analyzed by 2 h Europium cytotoxicity assays. Pooled data obtained with cells from three healthy PBMC donors and five B-ALL patients at the effector to target (E:T) ratios of 40:1 (left) and 20:1 (right) are shown. Bars and error bars represent means of results and standard deviations, respectively. ns: not significant; *: significant (p-value < 0.05).

Supplementary Tables

Table S1. Clinical characteristics of B-ALL patients and FLT3 surface expression levels.

UPN	BCR-ABL[1]	ALL type	Risk	Age	Sex	WBC [G/L]	Hb [g/dL]	Plt [G/L]	Karyotype[2]	MLLr[1]	% PB	% BM	SFI FLT3	% FLT3+	% CD20+	% CD19+	% CD22+	% CD34+	% CD10+
1	-	pro B	HR	63	m	39.97	10.1	125	46,XY	+	96	96	27.6	98	1	92	53	59	0
2	-	pro B	HR	36	f	51.99	12.4	247	48,XX,+Xor+7,t(4;11)(q21:q23),+der(4)t(4;11)(q21;q23),+22/46,XX	+	79	80	12.3	68	3	88	24	2	1
3	-	common	HR	24	m	38.63	7.3	76	n.a.	n.a.	n.a.	n.a.	10.9	35	8	64	21	90	28
4	-	common	HR	21	m	28.27	11.7	280	n.a.	n.a.	92	n.a.	92.6	100	0	100	86	100	30
5	-	common	HR	31	f	14.23	5.4	80	n.a.	-	68	79	1.1	10	9	80	70	63	4
6	-	common	HR	36	m	33.59	5.0	11	46,XY	n.a.	92	95	8.3	83	7	92	82	88	71
7	-	common	SR	33	m	13.46	13.0	155	46,XY	-	67	100	1.6	21	16	90	76	3	73
8	-	common	SR	61	m	7.87	8.5	21	46,XY	n.a.	54	n.a.	34.0	85	n.a.	n.a.	n.a.	n.a.	n.a.
9	-	pre B	HR	22	m	74.14	13.2	13	46,XY	n.a.	n.a.	n.a.	12.0	49	n.a.	n.a.	n.a.	n.a.	n.a.
10	-	pre B	HR	61	f	123.81	8.0	40	n.a.	-	98	97	72.2	96	17	100	81	80	0
11	-	pre B	SR	45	m	10.55	9.4	21	46,XY	n.a.	66	98	10.0	87	10	77	70	57	43
12	-	pre B	SR	24	f	29.22	10.3	33	46,XX	n.a.	86	97	65.5	97	12	84	82	67	25
13	-	pre B	SR	41	m	6.81	9.3	44	46,XY	n.a.	38	84	1.0	1	n.a.	n.a.	n.a.	n.a.	n.a.
14	+	common	VHR	50	f	364.80	8.3	30	48,XX,t(9;22)(q34;q11),+der(22)t(9;22)(q34;q11),+C,?inc	-	97	n.a.	10.7	49	8	n.a.	13	82	n.a.
15	+	common	VHR	76	m	68.75	9.6	20	47,XY,t(2;16)(p11;p11),+der(8)t(8;8)(p23;q23),der(8)t(8;8)(p23;q23), t(9;22)(q34;q11)/48,XY,+X,t(2;16)(p11;p11)der(8)t(8;8)(p23;q23), t(9;22)(q34;q11)+der(22)t(9;22)(q34;q11)/46,XY	-	82	n.a.	30.9	87	45	86	87	27	80
16	+	common	VHR	64	m	30.01	13.6	141	46,XY,t(9;22)(q34;p11)/46,XY	n.a.	57	n.a.	1.8	15	11	73	40	75	60
17	+	common	VHR	81	f	114.17	8.8	35	46,XX	n.a.	97	n.a.	69.3	95	7	97	30	96	93
18	+	common	VHR	25	m	4.69	4.8	15	46,XY	-	60	81	16.1	94	30	66	23	53	56
19	+	common	VHR	21	m	463.01	11.6	31	n.a.	n.a.	87	93	7.2	84	66	89	52	52	91
20	+	common	VHR	49	f	56.81	10.3	48	46,XX	n.a.	92	94	19.6	35	13	91	50	82	88
21	+	common	VHR	32	f	19.37	7.2	170	n.a.	n.a.	56	98	19.9	80	54	82	88	80	79
22	+	common	VHR	49	m	222.77	12.3	40	46,XY,add(5)/p1?5)5,del(6)(q2?3),del(7)(p1?5),del(7)(q?22), del(9)(q?22),t(9;22)(q34;q11),add(12)(p1?),del(13)(q12q?22), add(19)(q13.?),?inc[cp23]/46,XY	-	94	n.a.	6.4	76	9	96	55	78	94

[1] assessed by PCR or FISH; [2] assessed by classical cytogenetics. UPN: uniform patient number; BCR: breakpoint cluster region; ABL: Abelson murine leukemia viral oncogene homolog 1; −: negative; +: positive; ALL: acute lymphoblastic leukemia; SR: standard risk; HR: high risk; VHR: very high risk; f: female; m: male; WBC: white blood count; G/L: Giga per liter; Hb: hemoglobin; Plt: platelets; MLLr: mixed-lineage leukemia rearrangement; n.a.: not available or not applicable; PB: peripheral blood blasts among nucleated cells; BM: bone marrow blasts; SFI: specific fluorescence intensity.

© 2019 by the authors. Licensee MDPI, Basel, Switzerland. This article is an open access article distributed under the terms and conditions of the Creative Commons Attribution (CC BY) license (http://creativecommons.org/licenses/by/4.0/).
